# Augmented reality flavor: cross-modal mapping across gustation, olfaction, and vision

**DOI:** 10.1007/s11042-021-11321-0

**Published:** 2021-09-06

**Authors:** Osama Halabi, Mohammad Saleh

**Affiliations:** Department of Computer Science and Engineering, Qatar University, Doha, Qatar

**Keywords:** Augmented reality, Olfactory display, Gustation, Cross-modal mapping, Flavor

## Abstract

Gustatory display research is still in its infancy despite being one of the essential everyday senses that human practice while eating and drinking. Indeed, the most important and frequent tasks that our brain deals with every day are foraging and feeding. The recent studies by psychologists and cognitive neuroscientist revealed how complex multisensory rely on the integration of cues from all the human senses in any flavor experiences. The perception of flavor is multisensory and involves combinations of gustatory and olfactory stimuli. The cross-modal mapping between these modalities needs to be more explored in the virtual environment and simulation, especially in liquid food. In this paper, we present a customized wearable Augmented Reality (AR) system and olfaction display to study the effect of vision and olfaction on the gustatory sense. A user experiment and extensive analysis conducted to study the influence of each stimulus on the overall flavor, including other factors like age, previous experience in Virtual Reality (VR)/AR, and beverage consumption. The result showed that smell contributes strongly to the flavor with less contribution to the vision. However, the combination of these stimuli can deliver richer experience and a higher belief rate. Beverage consumption had a significant effect on the flavor belief rate. Experience is correlated with stimulus and age is correlated with belief rate, and both indirectly affected the belief rate.

## Introduction

People’s interaction with interfaces has mostly been limited to visual, and to a lesser extent, auditory inputs. Recent progress in human interfaces starts to include other sensory modalities such as touch or haptic and to less extent olfaction. It is more likely that human–computer interaction in the next years will incorporate more interaction modalities such as touch and olfaction. This will likely increase the engagement of people and make them more connected and deliver a richer online experience in many domains such as education, entertainment, shopping, training, etc. [26]. With the COVID-19 pandemic lockdown, the need for such rich interaction becomes inevitable and pave the way for a new digital interaction era.

Recent advancements in Virtual Reality and Augmented Reality enabled the development of an immersive environment with a higher sense of realism [10, 14]. Sound and 3D graphics are presented to create a sense of an artificial environment. AR system was proposed to provide new food experience eating by generating different food images “DeepTaste” [23]. The Image can affect the taste perception [2, 20] Sometimes haptic interface is used to enable users to physically interact with the virtual environment (VE) through touching the 3D objects [11–13]. However, adding olfactory and gustatory senses is still in its infancy. This is due to the difficulty in dealing with these two senses as they are based on the chemical signal, meanwhile visual, auditory, and haptic senses are physical signals. Therefore, there have been few studies that explored gustatory display [17, 25]. Combining AR technology with olfactory display has the potential in the food domain where it can improve people’s eating experience, especially for those who can eat limited kinds of foods due to their health problems [43].

This work is to explore more in-depth how vision and olfactory can affect the gustatory sense by taking advantage of the cross-modal nature of flavor. This assumes that when ingesting an object, a combination of senses contributes to the “flavor”. Resultantly, perception of flavor can be said to be influenced by a combination of smell, touch, auditory cues, and visual cues.

It is generally established that aroma, taste, texture, and mouth feel account for the major stimuli that contribute to the perception of flavor [40]. The flavor sensation is influenced by other senses such as olfaction, vision, memory [8], and even sound [33]. The perception of drink or food is influenced by different cues from other sensory modalities. Many studies verified the multisensory properties where gustation is affected by vision and olfaction [16, 34–36]. The tendency of associating a pair of stimuli, objects, or events from different sensory modalities are referred to as cross-modal correspondences [37] which are used as criteria to evaluate if the pair is crossmodally congruent or not. The benefit of cross-modal congruency between two stimuli on many aspects has been demonstrated. It enhances identification or detection [19, 41], improves memory and learning [3, 22], and facilitate attention and inhabitation in short-term memory [4, 21]. Flavor involves the combination of gustatory and olfactory stimuli and can be considered as one of the most multisensory experiences we frequently deal with in our daily tasks [36]. It has been widely agreed that the sense of smell (or olfaction) contributes to the majority of the information to our experience [38]. It has been suggested that 80%-90% of the flavor of food comes from the nose [39], and we all experienced how we are not able to recognize the flavor of the food when having a stuffy nose, moreover we lose the enjoyment of the food. This is also evident in how people describe different odors. According to a study at the University of Otago [27], “65% of assessors gave ‘sweetness’ as an appropriate descriptor for the odor of vanillin, while 33% described the odor of hexatonic acid as being sour”. This means that people inherently associate certain odors with their respective tastes. In another study by Djordjevic et al*.* [7] in which participants were separated into two groups one of which they asked to taste a solution that was scented. The second group was asked to imagine odors when tasting an unscented solution. Both solutions were unflavored. Consequently, they concluded that the perception of flavor can be prompted with both physically present and imagined odors. This provides proof of the ability to effectively simulate the scent associated with a certain beverage flavor which is the core of this work.

In addition to olfaction, visual information is also an important factor to consider when attempting to take advantage of the cross-modal nature of taste. The vision is more directly related to olfaction. Thus, when smelling a certain odor, one’s natural reaction is to associate it with an image or color. For instance, if one were to walk into a supermarket and smell a strawberry scent their eyes are drawn to red objects in an attempt to find the source of the odor. This association was established via an experiment conducted by Demattè et al. [5]; in their study, they took a group of 21 university students and had them smell six different odorants (caramel, cucumber, leather, lemon, spearmint, and strawberry). The participants were exposed to scent for 4 s and then shown 10 different colors (red, yellow, blue, orange, pink, brown, turquoise, purple, and grey) on a computer screen that remained until the participant picked one. Their instructions were to pick the color that was most closely associated with the odor. The experiment concluded that each odor was distinctly associated with a minimum of one color. Consequently, we can hypothesize that objects should appear and smell like a simulated object to be able to induce the desired flavor.

## Related work

It is difficult to build a gustatory display due to the complexity of the cognition mechanism involved in gustatory sensation. Few studies explored gustatory display where VR and AR were used to present the visual element of the food. Wang et al. explored how altering the color of a coffee in VR influenced the flavor perception of cold-brew coffee [42], Aoyama et al. also introduced a method to generate galvanic taste in VR/AR simulation to modify taste sensations and support easting restriction systems [1]. However, both previous works deal only with vision and no smell element. Kerruish [18] presented a review paper on the importance of integrating smell in VR and AR to simulate the real world and introduced Vocktail [30] and Season Traveler [28] as two good examples of digital devices that incorporate taste and smell. Season Traveler provides a wearable Head Mounted Display (HMD) system that can deliver smell, thermal, and wind stimuli when users explore different landscapes virtually. Petit et al. discussed in their review paper the new opportunities of integrating digital sensory in a new multisensory online experience with the addition of haptic and olfaction [26]. Narumi et al. [24, 25] presented a pseudo-gustatory display by overlaying visual and olfactory information on a plain (not flavored) cookie to make it appear like several digitally different manipulated versions of flavored cookie. In 72.6% of the trials, the participants identified the taste of the augmented cookies correctly. The previous work studied the flavor in solid food as in cookies, meanwhile, the main focus of this research is to explore the cross-modality in liquid food such as drinks as it presents different characteristics and challenges as mentioned before. Besides, the analysis was simple and no proper scientific statistical analysis was presented to verify the significance of the results. The work introduced in [29, 31], a digital instrument to stimulate taste sensation digitally using electrical and thermal stimulation on the tongue as well as smell. Preliminary experiments showed that users were able to distinguish different flavors such as spicy, minty, and lemony. The smell improved the taste sensation at an average of 62% to 83%. However, visuals were not considered in the study. The work in [30] presented a virtual cocktail system that digitally simulated multisensory flavor experience that utilized three sensory modalities. The combination of taste, smell, and color delivered a richer flavor experience. As individual modalities, the color scored the lowest, the electric stimuli and smell scored the highest but still less than the combined set. However, they only used simple color stimuli and not a complex replacement of the visual and this limited the result, regarding the visual contribution to the overall taste sensation. Besides, the work only explored the taste and not the flavor.

This study presents a customized wearable AR display with olfaction display using VR HMD to demonstrate gustatory display for liquids and to explore the contribution of each sense of the interaction modal to the final flavor decision. Moreover, factors such as age, previous experience in VR/AR, and beverage consumption are considered in the study. In this work, a depth exploration of the cross-modal association between the vision, olfaction, and flavor modalities to reveal how the perceived flavor is influenced by each modality. Moreover, similar previous works were experienced with only solid food and not liquid food. therefore, it is worthy to explore how AR gustatory display can induce flavors in liquid food as this can be utilized in the huge beverage industry.

## Gustatory display

**Fig. 1:**
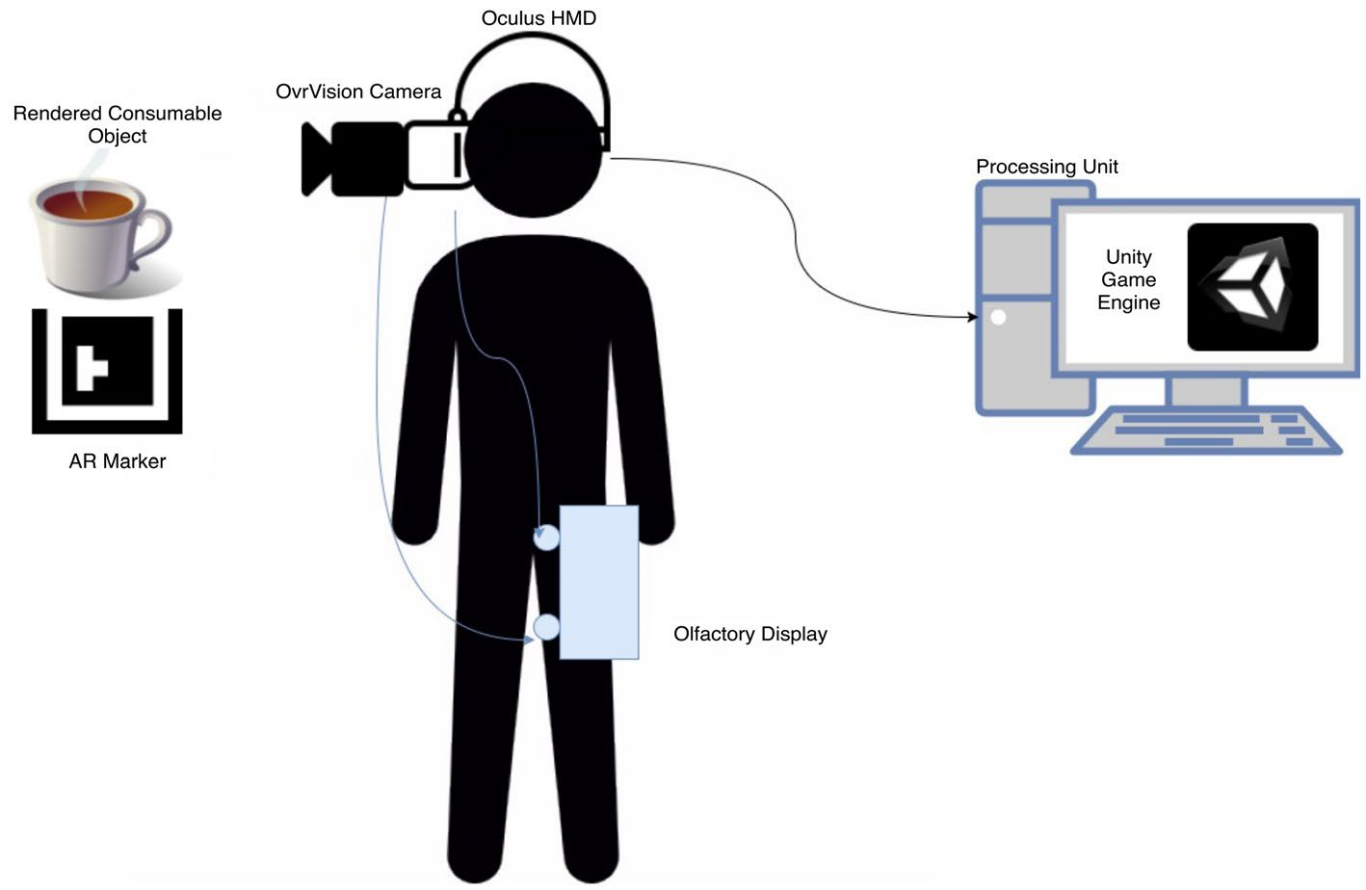
Overview of the system and its main components

In general, we need two displays: the first one is the AR display to superimpose the images on real objects, and the second display is the olfactory display to produce the sense of smell. Each display type can be developed using different technologies. For example, AR displays can use different AR glasses which seems a better option, however, the price is expensive and the resolution is less while the proposed solution is low-cost and affordable. As for the olfactory display, it is based on the solid model developed and tested in previous work [9, 8]. The proposed system is composed of these two main components; the AR sub-system to produce the intended images and superimpose them on the real object, and the olfactory display. An overview of the system can be seen in Fig. 1.

### Visual component

The visual component can be considered the core of the experiment as most of the processing happens within it. It consists of three subcomponents: processing device, video camera, and HMD. The steps carried out for visual generation are as follows:

- The Video camera takes input from the surroundings and passes the input over to the processing unit.
- The processing unit processes the video feed in an attempt to locate the AR marker located in the surrounding environment.
- Once the AR marker is located, the Unity game engine generates a game scene with the aid of the OvrVision SDK which contains the desired changes to the augmented reality world.
- The modified video feed is then passed over to the display (HMD) which then displays the modified video feed to the user who is wearing the HMD.

The roles of the visual sub-system include:

- The detection of the original consumable object and the generation and placement of the image that will sit atop the consumable object in the augmented reality environment.
- Signaling the olfactory display when it detects the consumable object moving closer towards the user’s mouth.
- The generation of visual distraction.
- The generation of the auditory distraction.

### Olfactory display

**Fig. 2:**
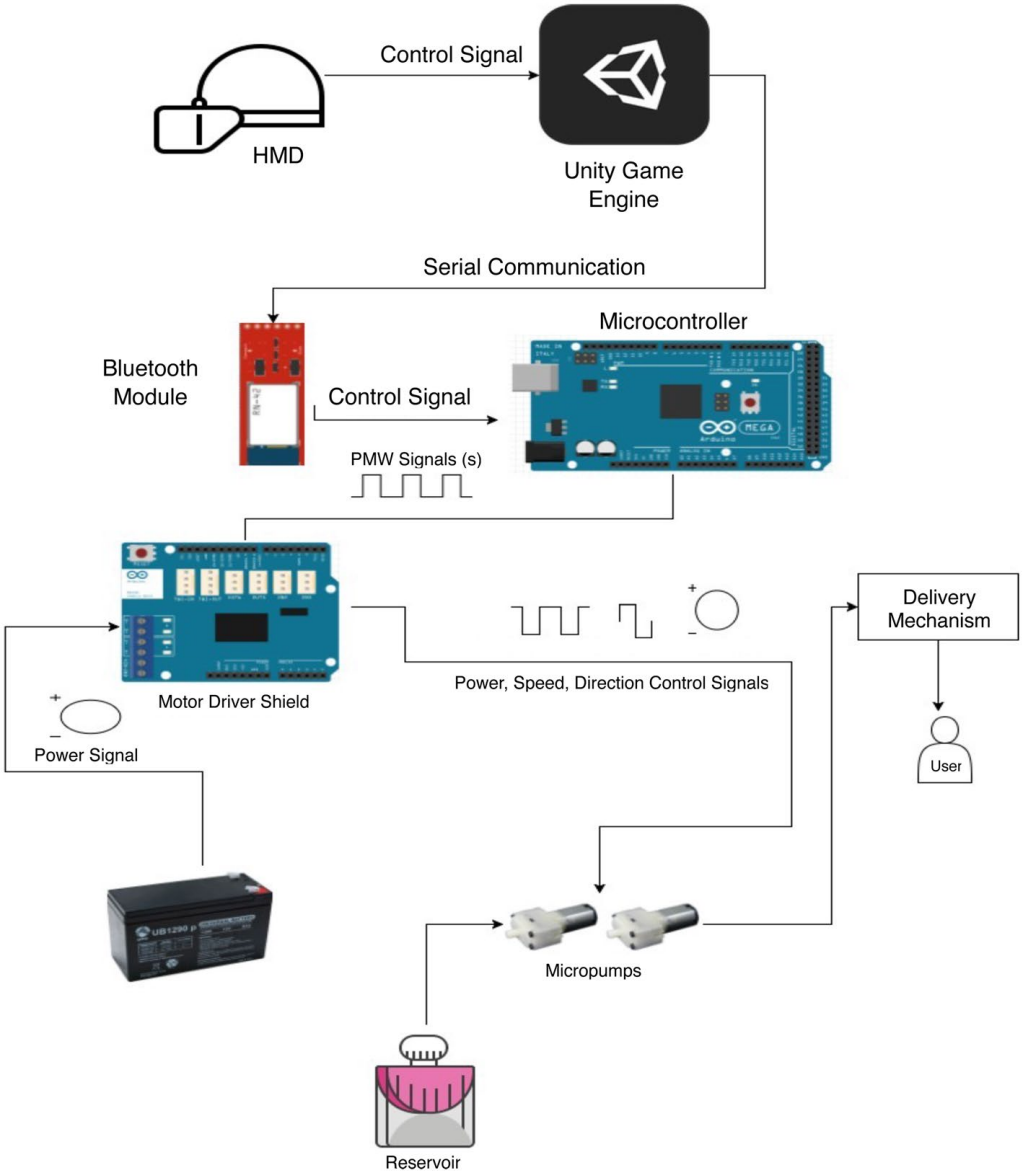
The hardware diagram and the components of the olfactory display

The Olfactory display was improved from previous work [9] with sufficient air flow rate and a new delivery mechanism using check valves to ensure no back leaking of the air, in addition to seamless integration with the latest version of VR HMD Oculus Rift, see Fig. 4. The hardware design consists of Arduino Mega 2560 microcontroller to produces the control signals for the level shifter, RF amplifier, and Motor Driver. It is responsible for processing the inputs to the display from Unity via Bluetooth module and using them to produce outputs via way of the Arduino Motor Shield to control each air-pump. The scent diffuser consists of two DC Micro air pumps (AJK-B06A2701), airline tubing to guide and deliver the air, and a check valve was used as small reservoirs of cotton balls soaked in liquid fragrance. The hardware components and their logic diagram are shown in Fig. 2.

**Fig. 3:**
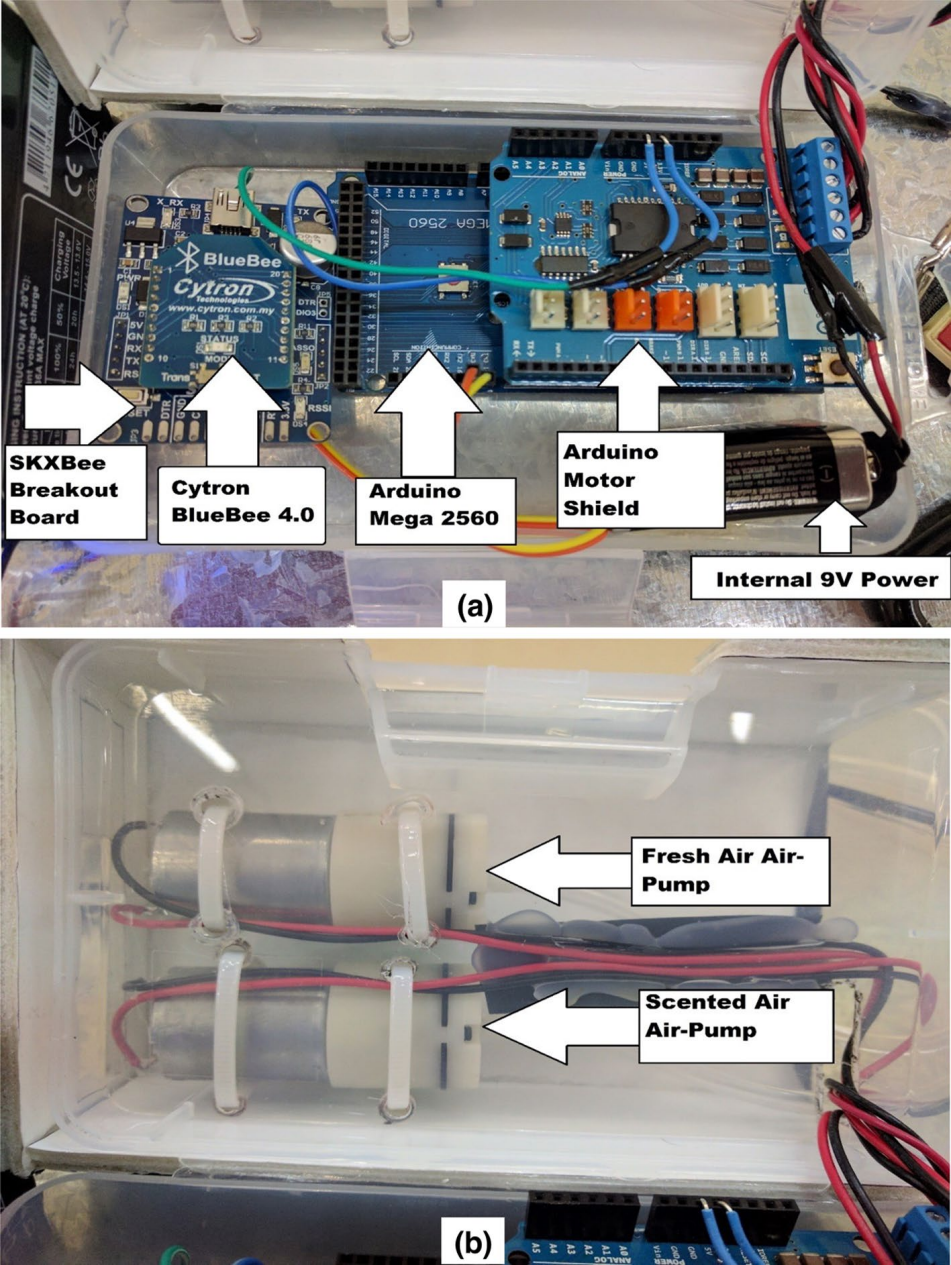
The internal view of the hardware components of the olfactory display assembled in two plastic boxes. The assembled hardware with power box (**a**), and the air pumps box (**b**)

The hardware components were assembled in two small compact boxes that made portable where the user can hang on his belt without distraction as can be seen in Fig. 5. The hardware internal view of the whole display is shown in Fig. 3.

**Fig. 4:**
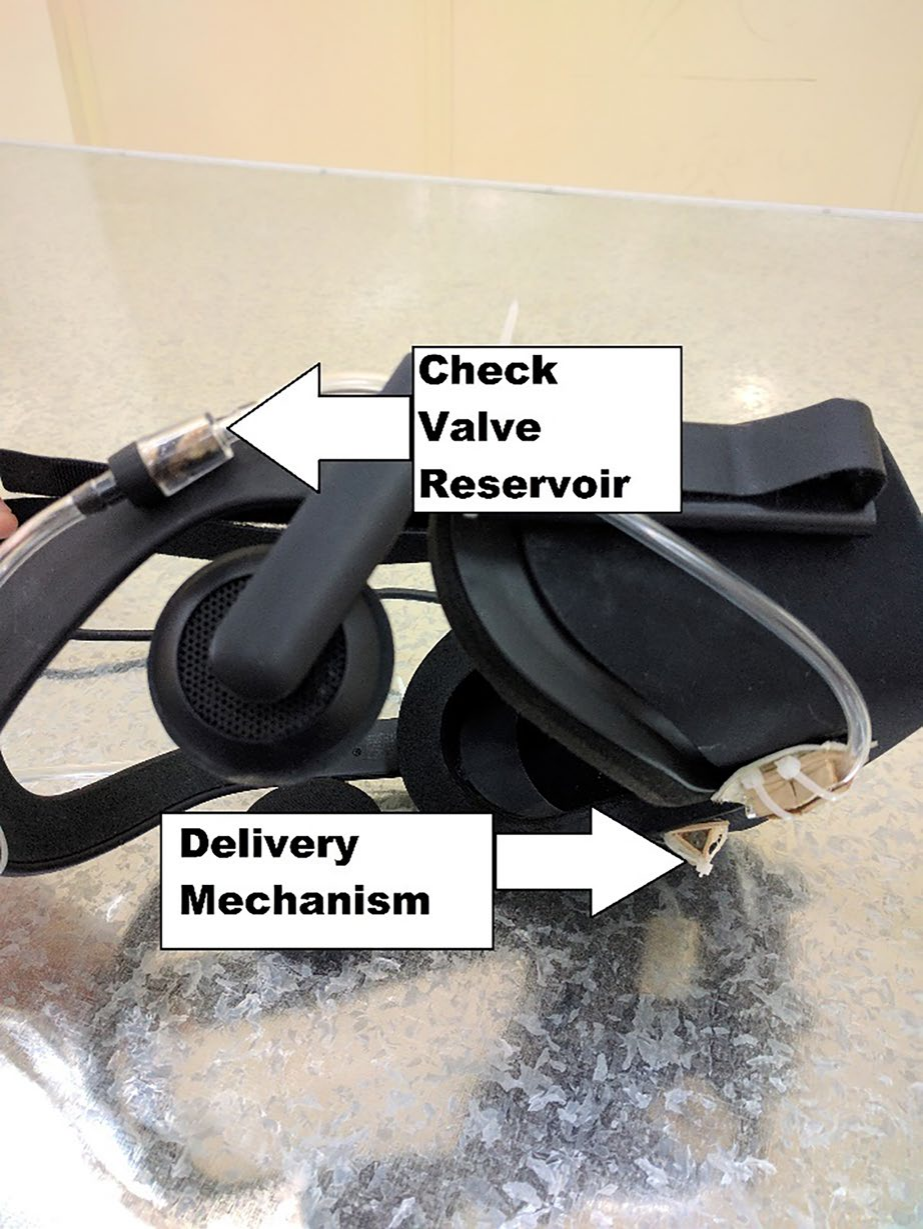
Smell delivery mechanism with HMD

**Fig. 5:**
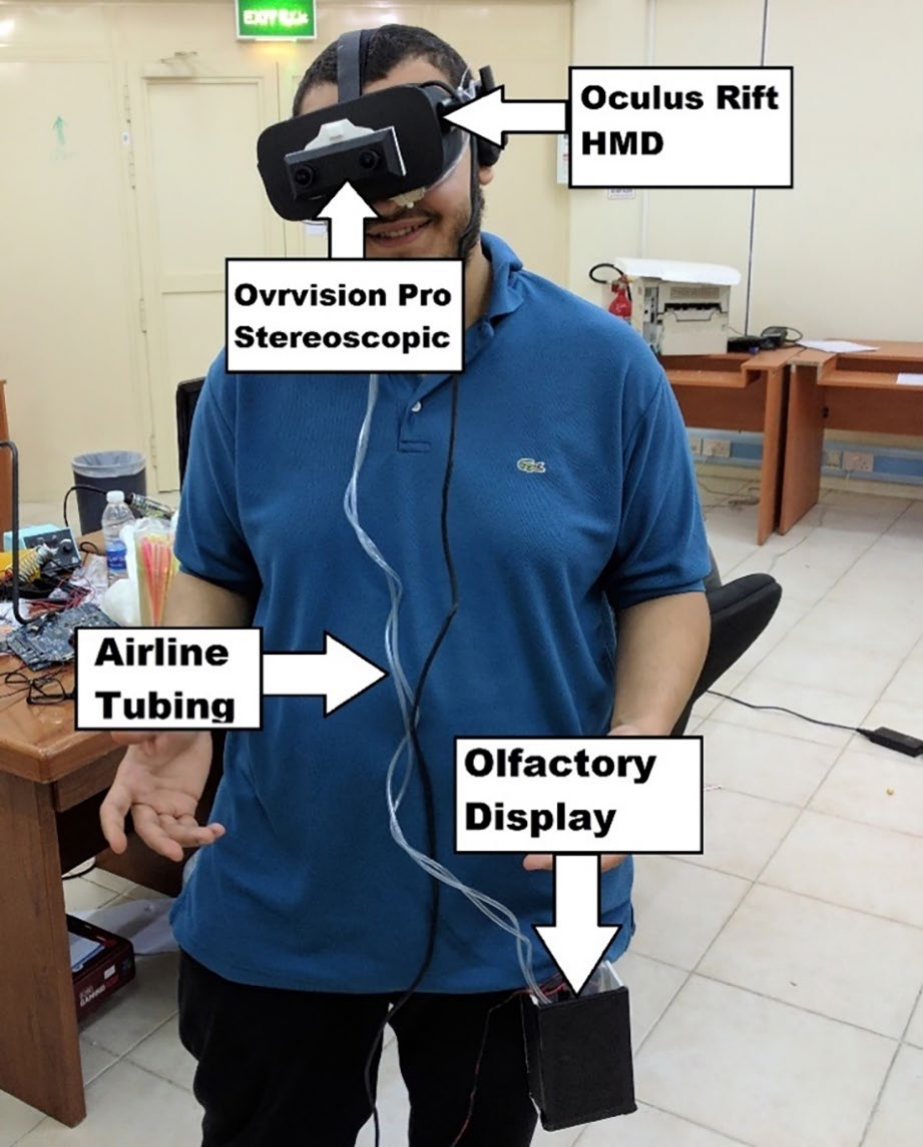
All the system components modelled on the user

The olfactory display is programmed to wait in idle mode until it receives a signal from the visual sub-system, which indicates that the consumable object is detected by the AR headset. When the object is detected the olfactory display begins to expel scented air until it receives another signal indicating that the consumable object had moved away from the subject’s nose. When this second signal is received, the olfactory display expels fresh scentless air to refresh the air. The air delivery mechanism consists of air tubbing to guide the air through a check valve to the user or directly to the user. Thus, the user is provided with either scentless fresh-air to dissipate any lingering scents or scented air, see Fig. 4. The whole system component modeled on the user is shown in Fig. 5.

### Augmented reality display

Oculus Rift was used as a headset to deliver the vision. However, since it is designed for VR, OvrVision stereo camera was fixed on the HMD to generate the AR environment, see Fig. 5. The camera act as eyes into the real world. When the marker is detected AR scene is rendered and signals are sent to the olfactory display notifying to start the smell induction phase. Since the objective is to augment the cup with different images to simulate different beverage flavors, the cup was detected using a marker on the bottom of it and fine adjustments were required to superimpose the image on the top of the cup. The fine alignment was critical to not destroy the realism of the beverage texture.

## Experimental method

Since many studies suggested that the gustatory system is optimally tuned to complex stimuli that engage several of senses simultaneously [32]. Therefore, the main objective of the experiments is to study the cross-modal association between vision, olfaction, and gustation. Moreover, a recent study showed that multisensory convergence occurs in brainstems at the very earliest stages of central processing of food. Teste, texture, and temperature information are derived from the oropharynx, but olfactory originated from top-down input and may be conveyed via the gustatory cortex, amygdala, or lateral hypothalamus, which means it happens at early stage that can enable rapid identification and ingestion or rejection decisions to be made [6]. The previous finding from a neural study perspective, therefore, it will interesting to investigate the previous finding and see how texture and smell information contribute to the final gustatory decision quantitively.

The hypothesis is that a combination of multi-sensory can make the subjects believe that a flavorless liquid with no taste such as Coffee-Mate creamer can taste like coffee if augmented with relevant texture and smell. To analysis, the effect of each sense on the final perceived flavor, the experiment conducted in three settings of senses: (1) only vision was presented and no olfaction (Vision set), (2) only smell was presented and vision was disabled (Olfaction set). (3) both vision and olfaction were presented (Combined set). A between-subject design is used to test the independent variables. An In-between subject methodology was used to assure no learning transfer or previous experience with the flavor. Thirty subjects were recruited per experiment to evaluate the targeted flavor with a total of 90 subjects for all experiments. The subjects were mainly engineering students, faculty, lab instructors, and family members. This design assured that the subjects will not learn about each condition and will not know what is the real flavor at the beginning of each experiment. Demographic information of the participants, consumption of coffee, the experience of VR/AR, along with their opinions were collected using the online questionnaire. Each subject was simply given a cup of Coffee-Mate creamer after wearing the system and asked to decide the tasted flavor freely without any given preferences or suggestions for any flavors. The result was recorded as correct/incorrect. Additional three independent variables were considered in the study that related to age, previous experience in VR/AR, and the level of coffee consumption to have a comprehensive analysis as these factors may contribute to the result and affect the participants' belief decisions. The age variable was divided into three categories, (17–22), (23–28), and (above 28). The experience in VR/AR variable was divided into four categories (novice, tried AR, tried VR, and tried both). Finally, the coffee consumption was also divided into four categories (occasionally, 2–3 times a week, Almost every day, and more than 3 times daily).

### Data analysis

The data were normalized and the mean score for each experiment set was used in visualization. A factorial ANOVA with Tukey post-hoc correction at an alpha level of 0.05 was applied to analyze the result of the accuracy of detecting the taste perceived in the three stimuli experimental settings. The dependent variable was the belief of flavor is coffee, while the four independent variables were stimulus, age, experience, and coffee consumption level. To further study the interaction between the different factors on the belief rate, Spearman's Rank-Order Correlation is used to explore the association that exists among all factors.

### Data distribution

**Fig. 6:**
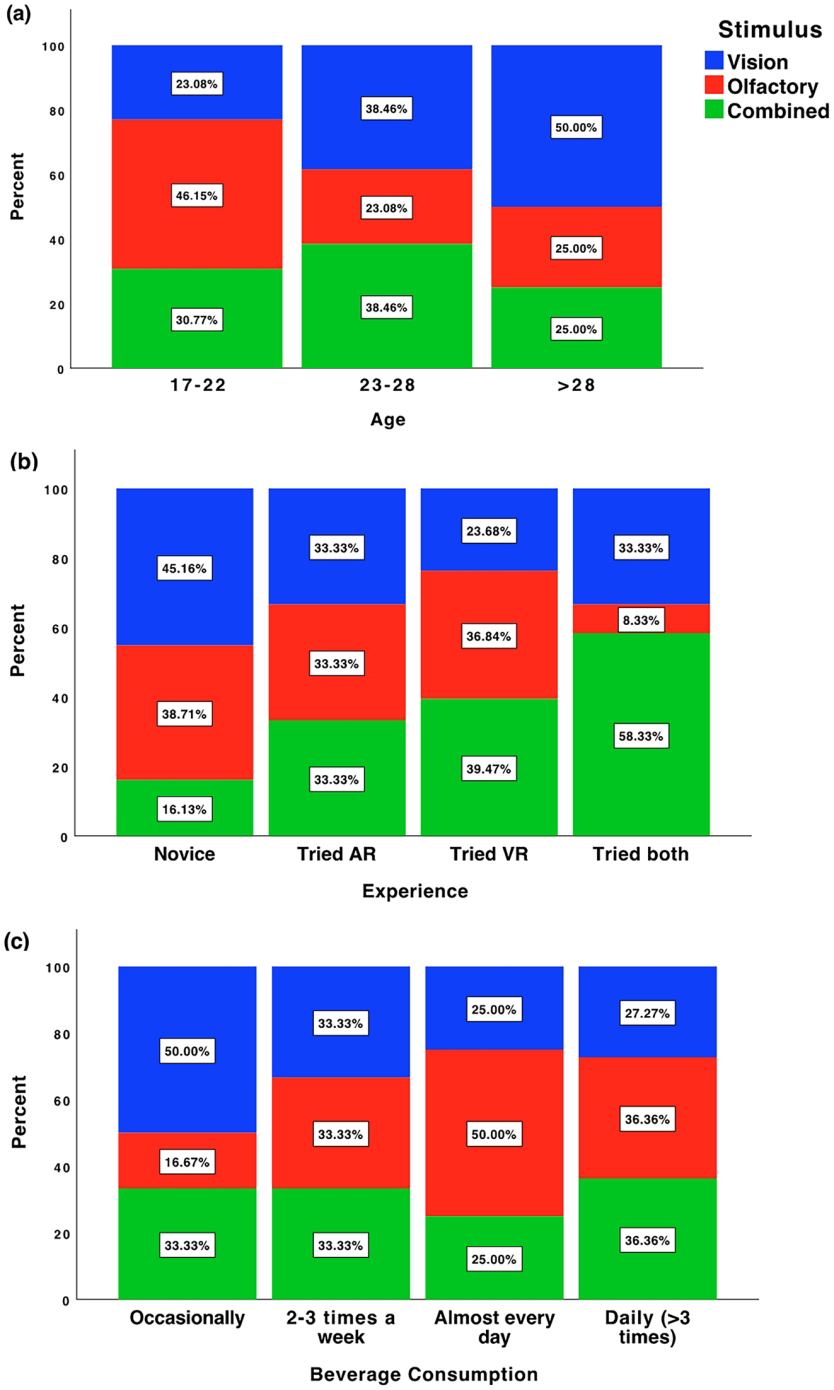
The distribution of different factors across each experimental setting. The age factor (**a**), the experience factor (**b**), and the consumption factor (**c**)

The subjects are different in each stimulus mode since the experimental design was in-between subjects design. Therefore, it was essential to check the distribution of the factors among the three settings to assure balance and make sure that each factor has a representative in each experiment mode. Figure 6 shows the distribution of each factor in each interaction mode. Generally, we can confirm that each stimulus mode has a representative for each factor. Each factor has a fair percent of subjects above 25%, and only in Olfaction set has small percent in tried VR criteria related to experience factor (8.33%), see Fig. 6b and 16.67% in “occasionally” condition related to beverage consumption, see Fig. 6c. The percentage of novice users in combined mode is also 16.67%, otherwise, the data have a good balance between all conditions. The subjects were asked about beverage consumption to address different consumption ratio by the subjects. It was evident that most subjects drink coffee frequently and only less than 30% do not drink coffee which makes the majority of subjects are familiar with the coffee taste and has an authenticate judgment.

### Experimental procedure

To test the theory that gustation can be manipulated by olfaction and vision we devised three experiments that were conducted with the proposed system to test the hypothesis that olfaction enhances the gustation and combined multimodal interaction of vision and olfaction has better performance than vision or olfaction alone.

### Vision only

In this experiment, the smell was disabled and only vision was presented. So, the olfactory display will be turned off during this experiment, but the appearance of the creamer will be digitally altered to that of coffee. Thus, the user only saw coffee.

### Olfaction only

In this experiment, the only smell was presented to aid in evaluating the flavor. Although no vision is presented, the subjects are asked to wear the device and the black image represents darkness. The subject has to rely only on the sense of smell to judge the taste.

### Vision and olfaction

This experiment was designed to test with both vision and olfaction enabled (Combined). In other words, the olfactory display was active during this experiment as well as the appearance of the creamer being digitally altered to that of coffee. Thus, the user saw and smelled coffee.

## Results

**Fig. 7:**
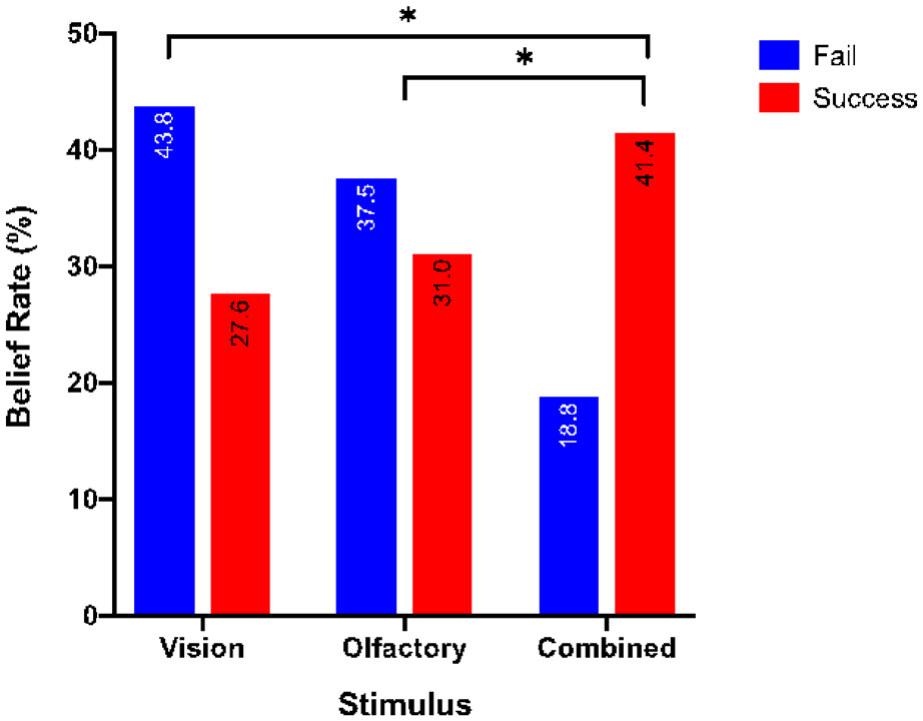
The overall belief rate for each stimulus mode

The One-way ANOVA analysis revealed a significant difference among the three stimuli sets with F (2, 57) = 4.851, p = 0.11. A post hoc Tukey analysis demonstrates that the belief score of the Combined set (success % = 41.4) was significantly higher than both the Vision set (success % = 27.6), p = 0.002, and 2), and the Olfactory set (success % = 31%), p = 0.028. No significant difference was found between the Vision set and the Olfactory set, see Fig. 7. This result demonstrated that combining sensory of Vision and Smell increases the gustation and made subjects more likely to believe that coffee creamer taste coffee. The overall success rate is low if compared to 72.6% in [22] study, however, this study is different as it is done on liquid as aforementioned and not on solid food. According to [40] and based on Harrison et al. study [15], diffusion of flavor compounds between lipid and aqueous phases is extremely rapid in liquid foods which will affect the release of flavor. This may explain the difference in the result. Moreover, only one flavor was tested, having a set of flavors to test might induce different responses and results.

### Age factor

**Fig. 8:**
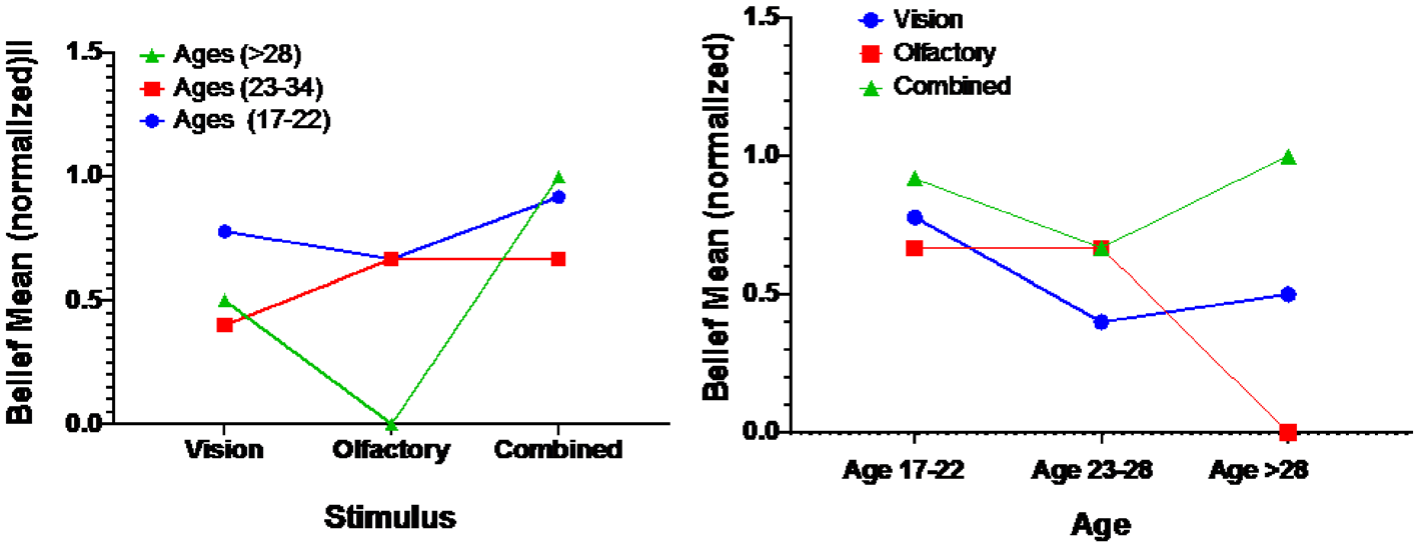
The belief rate in each stimulus mode for each age category (left), The belief rate for each age category in correspondence to each stimulus mode (right)

An interaction between stimulus and age was demonstrated, F(1,57) = 21.753, p = 0.000. A Post hoc Tukey analysis demonstrated that the belief score of the Age (17–22) set was significantly higher than both the Age (23–28) set (Mean Difference = 0.21), p = 0.009, and the Age (> 28) set (MD = 0.27), p = 0.020. No significant difference was found between the Age (23–28) set and the Age (> 28) set. Figure 8 (left) demonstrates that Age (17–22) performed higher than the other two groups, and the worst performance was for ages that are > 28. Figure 8 (right) shows clearly that the highest belief score was achieved in Combined mode in all ages sets, less in Vision, and the worst score was for the Olfactory set.

### Experience factor

**Fig. 9:**
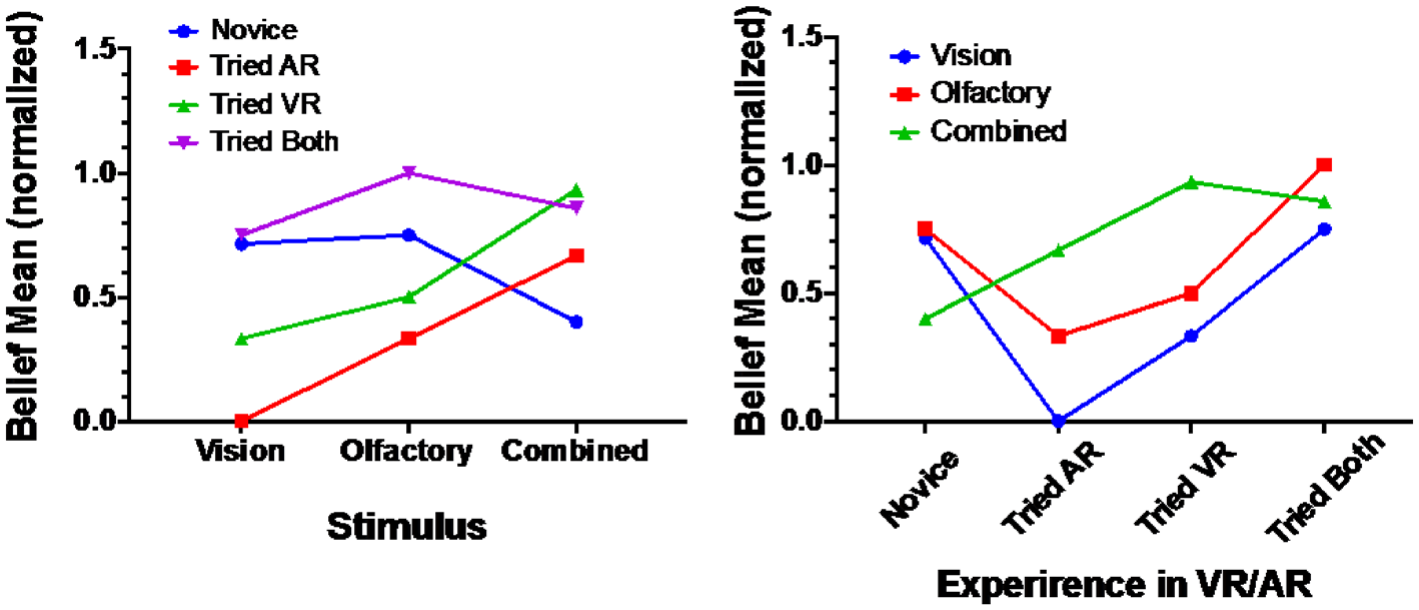
The belief rate in each stimulus mode for each experience category (left), The belief rate for each experience category in correspondence to each stimulus mode (right)

An interaction between stimulus and experience was demonstrated, F(2,57) = 8.720, p = 0.000. A Post hoc Tukey analysis revealed that the belief score of the Experience Novice set was significantly higher than the Tried AR set (MD = 0.34), p = 0.016. The Tired VR set was higher than the Tried AR set (MD = 0.50), p = 0.002. No significant difference was found between the Novice and Tried VR as well as Tried both. Figure 9 (left) demonstrates that Tried both group has the highest score than the other three groups, and the worst score was for Tried AR group. This comes as a surprise to what is expected, however, this might be because that the system is using a VR headset that blocks completely the surrounding vision and the camera are feeding the real-world images, wherein AR headsets or glasses usually the glasses are transparent and the images are fused into the real world view. The group that had experience in both VR and AR seems to be used to such an experience and felt comfortable, meanwhile, the AR only group used only see-through glasses and might felt different from their previous experience. Smilingly, the Novice group performed well as they might not expect the experience. Figure 9 (right) demonstrates clearly that the highest belief rate was achieved in the Combined set between all experience groups, Olfactory comes second, and the worst performance was in the Vision set.

### Beverage consumption factor

**Fig. 10:**
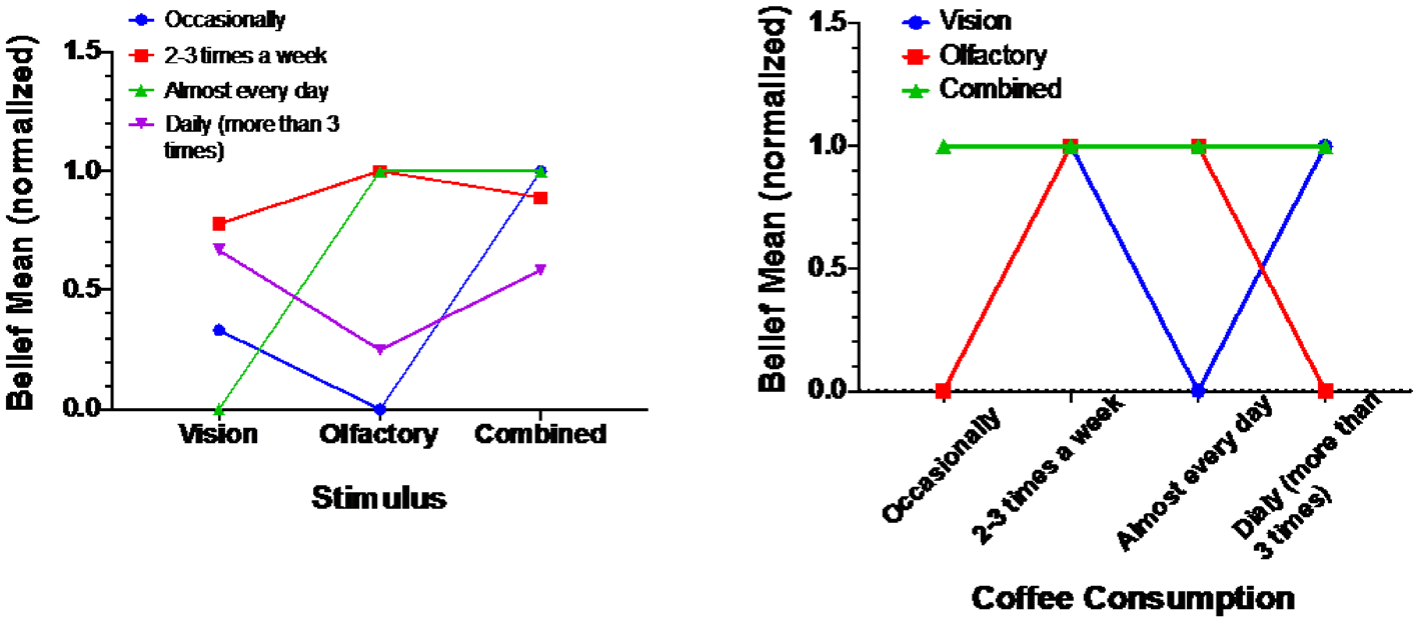
The belief rate in each stimulus mode for each consumption category (left), The belief rate for each consumption category in correspondence to each stimulus mode (right)

The analysis revealed a significant difference among the three Consumption sets with F(3, 57) = 6.073, *p* = 0.001. A Post hoc Tukey analysis revealed that the belief score of the 2–3 times a week set significantly higher than both the Occasionally set (MD = 0.39), *p* = 0.000, and the Daily set (MD = 0.27), *p* = 0.000. Also, Almost every day was higher than the Daily group (MD = 0.27), *p* = 0.46. Other relations were found to be of no significance. Figure 10 (left) demonstrates how 2–3 times a week group has the highest performance than the other three groups, and the worst performance was for the Occasionally group. Figure 10 (right) demonstrates clearly that the highest belief rate was also achieved in the Combined set, Olfactory comes second, and the worst performance was for the Vision set Fig. 10.

### Correlations

**Fig. 11:**
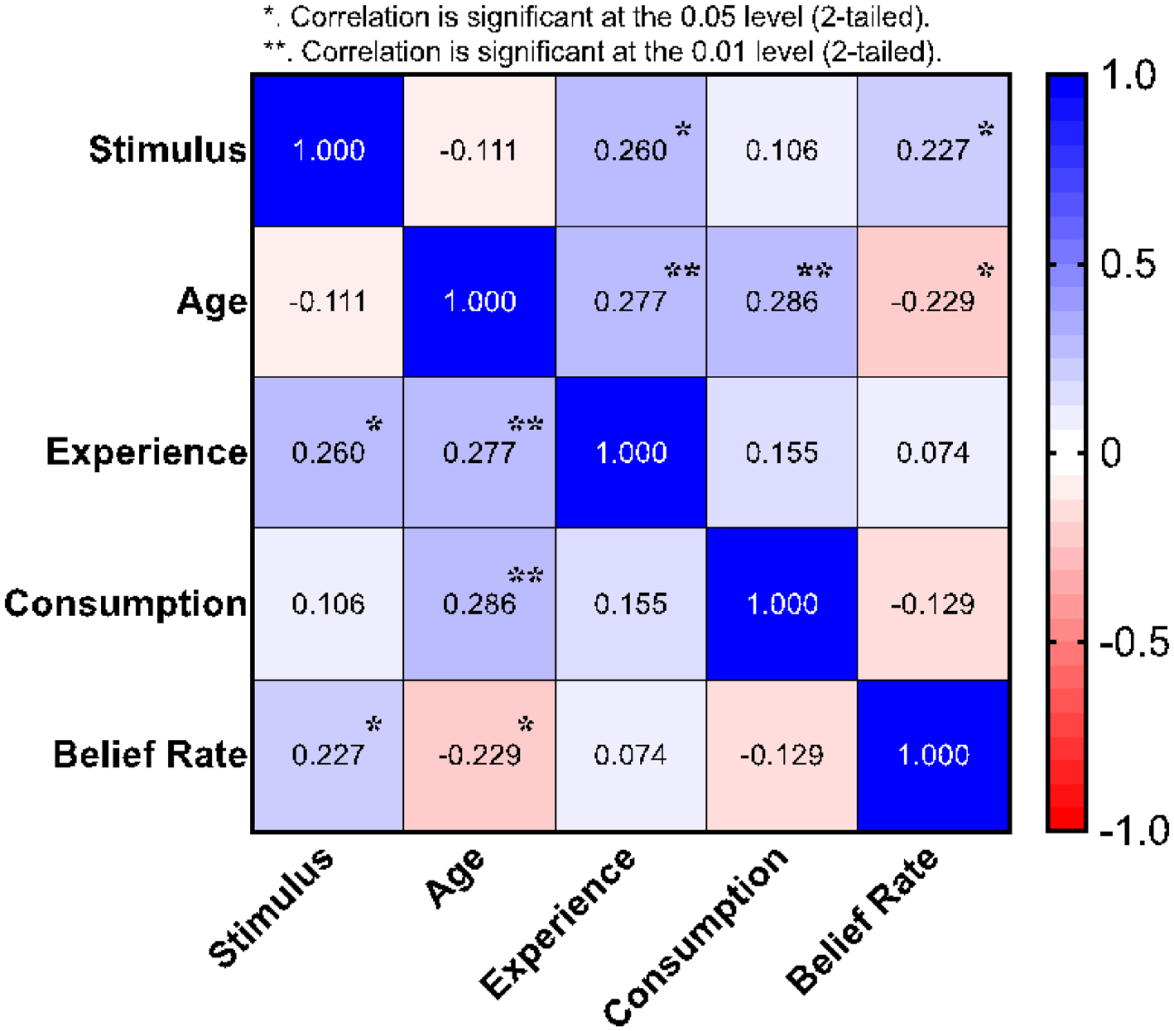
The heat map for correlations between all factors using Spearman's rank-order correlation, the values represents Spearman *r*_*s*_

**Fig. 12:**
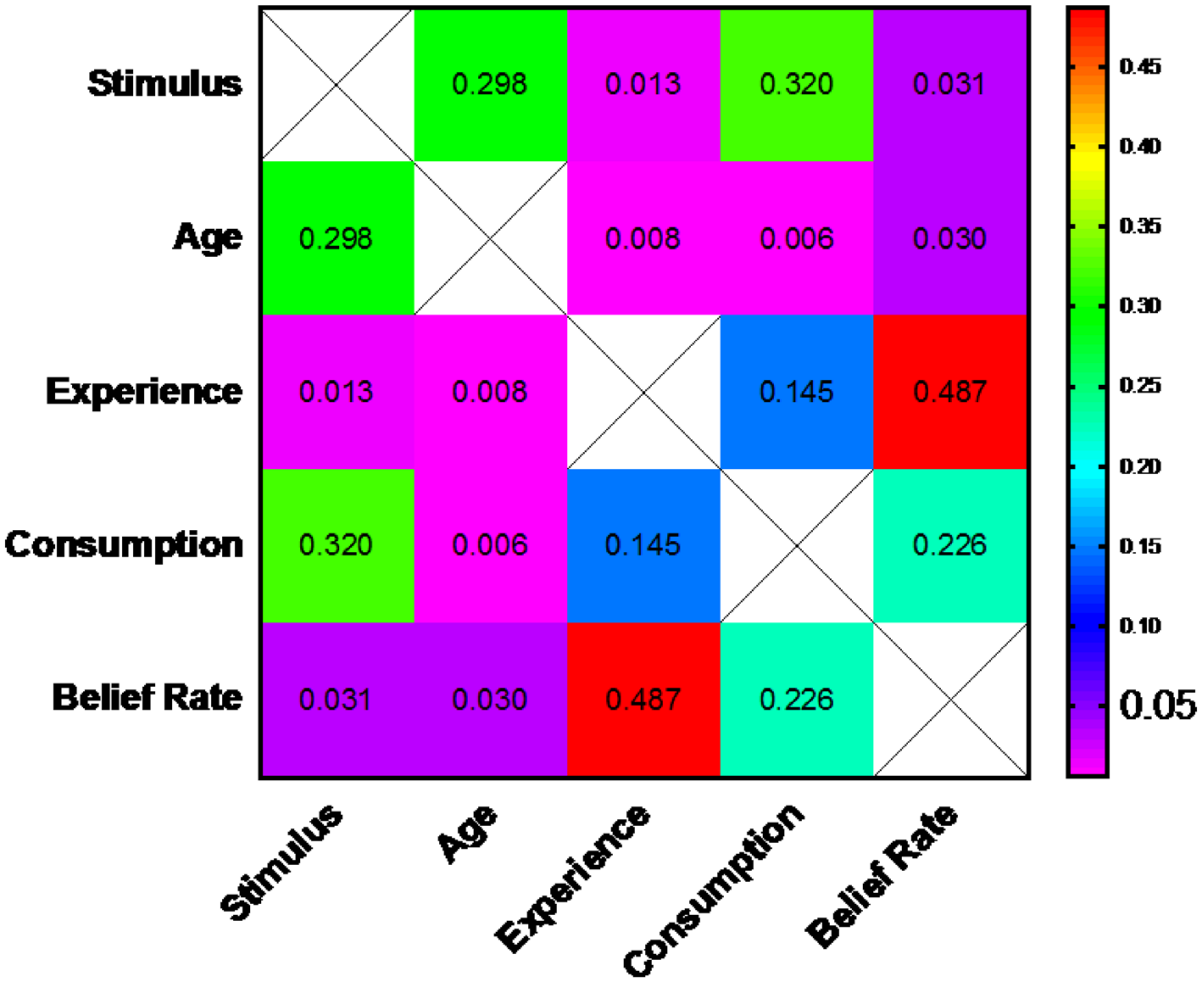
The p values heat map for correlations between all factors using Spearman's rank-order correlation

To study the correlations among all factors, a Spearman's rank-order correlation was run to determine the relationship between stimulus, age, experience, beverage consumption, and belief. This test is a nonparametric measure of the strength and direction of association that exists between variables so the normal distribution of the data is not a required assumption for this test. A heat map figure summarizes the results in Fig. 11 where it shows the Spearman *r*_*s*_ values for all factors. Figure 12 shows the p-value for every Spearman *r*_*s*_ relation to evaluate its significance. There was a weak, positive correlation between Age and 1) Experience, which was statistically significant (*r*_*s*_ = 0.256, *p* = 0.015), and 2) Consumption, which was statistically significant (*r*_*s*_ = 0.290, *p* = 0.006). This was expected since the elder has more experience and consume more beverage than youth. However, there was a weak, negative correlation between Age and Belief which was statistically significant (*r*_*s*_ = -0.222, *p* = 0.036). This means that as getting old the Belief rate decrease, therefore the Belief rate is higher in younger ages, which is also expected. There was a weak, positive correlation between Belief and Stimulus, which was statistically significant (*r*_*s*_ = 0.227, *p* = 0.031), which means that belief is the highest in the Combined set, then Olfactory set and Vision set comes last. This was also demonstrated in Figs. 8, 9 and 10.

## Discussion

The analysis of the experimental results of the AR flavor system revealed that interactions between smell, age, experience, and beverage consumption influenced the perception of flavor perception. The overall result for the tested hypothesis is that the Combined multisensory of smell and vision leads to the best results, where success rate increased by 13.8% compared with Vision alone, and 10% compared with Olfactory alone. The failing rate decreased 25% compared to that of Vision and 18.7% compared with Olfactory, see Fig. 7. This confirms the result of the aforementioned study [6] that the convergence of olfactory and gustatory information occurs in the early stages before texture.

In the analysis of belief rate for age, experience, and consumption, indeed the smell played a big role in increasing the belief of flavor perception. Age has a negative significant correlation with the belief rate, the elder the less accurate in judging the flavor. The experience affected the belief due to significant relation with stimulus. Having experience in both VR and AR increased the belief, therefore training and exposure to such new interaction media are important and it is essential to train the subjects before testing as this may affect the results. The consumption level of beverages had a significant effect on the belief rate, and this is normal as they are more familiar with the coffee taste and can provide an authentic decision.

The study explored in-depth the cross-modal mapping across vision, olfaction, and gustatory. However, there are few limitations to this study. The first one to mention is only one flavor has been tested and there is a need to include several other flavors to better confirm the result among any type of liquid. The study can be improved to explore in detail the five essential sensations of sweet, bitter, salty, sour, and umami.

## Conclusion

A system for inducing the flavor taste from the combination of smell and vision is presented in this paper. The technology is based on using AR to create the visual illusion of different beverages type and the proposed olfaction display was developed to present the sense of smell. An integrated system to combine both smell and vision was presented and tested. Experiments were carried out to explore the cross-modal association between smell, vision, and gustatory sense. The smell sense contributes vastly to the flavor taste. Vision can be a factor to assist or deceive depends on the shape and color of the substance. The study showed how olfaction plays a major role in gustation, which goes with the recent finding by neural studies [6] that olfactory occurs in the early stage via the gustatory cortex, amygdala, or lateral hypothalamus, which explain the rapid identification. This work can be further explored by addressing different types of liquids and consider further investigate the identification of the five essential tastes. Nevertheless, the combination of different sensation modalities can best improve the gustatory sensation. This work can motivate the future of flavor and smell interfaces to reach the next level of human–machine natural interaction by incorporating smell and taste. The virtual reality and gaming industry can most benefit from such interfaces in addition to innovative applications in the future. The food domain can benefit by increasing the food experience and find solutions to some eating problems.
